# Metformin in colorectal cancer: molecular mechanism, preclinical and clinical aspects

**DOI:** 10.1186/s13046-019-1495-2

**Published:** 2019-12-12

**Authors:** Muhamad Noor Alfarizal Kamarudin, Md. Moklesur Rahman Sarker, Jin-Rong Zhou, Ishwar Parhar

**Affiliations:** 1 Brain Research Institute Monash Sunway (BRIMS), Jeffrey Cheah School of Medicine and Health Sciences, Monash University Malaysia, 47500 Bandar Sunway, Selangor Malaysia; 2 Department of Pharmacy, State University of Bangladesh, 77 Satmasjid Road, Dhanmondi, Dhaka, 1205 Bangladesh; 3 Health Med Science Research Limited, 3/1 Block F, Lalmatia, Mohammadpur, Dhaka, 1207 Bangladesh; 4 Department of Surgery, Beth Israel Deaconess Medical Center, Harvard Medical School, Boston, MA 02215 USA

**Keywords:** Metformin, Colorectal cancer, Cancer, Type 2 diabetes mellitus, Chemopreventive, Anticancer

## Abstract

Growing evidence showed the increased prevalence of cancer incidents, particularly colorectal cancer, among type 2 diabetic mellitus patients. Antidiabetic medications such as, insulin, sulfonylureas, dipeptyl peptidase (DPP) 4 inhibitors and glucose-dependent insulinotropic peptide (GLP-1) analogues increased the additional risk of different cancers to diabetic patients. Conversely, metformin has drawn attention among physicians and researchers since its use as antidiabetic drug exhibited beneficial effect in the prevention and treatment of cancer in diabetic patients as well as an independent anticancer drug. This review aims to provide the comprehensive information on the use of metformin at preclinical and clinical stages among colorectal cancer patients. We highlight the efficacy of metformin as an anti-proliferative, chemopreventive, apoptosis inducing agent, adjuvant, and radio-chemosensitizer in various colorectal cancer models. This multifarious effects of metformin is largely attributed to its capability in modulating upstream and downstream molecular targets involved in apoptosis, autophagy, cell cycle, oxidative stress, inflammation, metabolic homeostasis, and epigenetic regulation. Moreover, the review highlights metformin intake and colorectal cancer risk based on different clinical and epidemiologic results from different gender and specific population background among diabetic and non-diabetic patients. The improved understanding of metformin as a potential chemotherapeutic drug or as neo-adjuvant will provide better information for it to be used globally as an affordable, well-tolerated, and effective anticancer agent for colorectal cancer.

## Background

Cancer remains one of the leading cause of death with high global prevalence despite numerous advancements made in the last decade. A recent cancer statistics by American Cancer Society projected a total of 1,762,450 new cancer cases with 606,880 mortality to occur in the United States alone [1]. The report estimated prostate (20%), lung and bronchus (13%), and colorectal (9%) to be the most prevalent new cancer cases in males whereas breast (30%), lung and bronchus (13%), and colorectal (8%) in females in 2019. Among this, respiratory and digestive system cancers are projected to contribute the highest mortality rate among other cancers. Colorectal or colon cancer (CRC) is projected to record the highest mortality cases (51,020) among other digestive system cancer (total of 165,460 cases) [1]. Factors such as bad dietary habits, smoking status, alcohol consumption, genetic predisposition, obesity, diabetes mellitus, and sedentary lifestyle significantly increase the risk of developing CRC [2–4]. To date, surgery, such as right colectomy, sigmoid colectomy, and total abdominal colectomy with ileorectal anastomosis as well as chemotherapy are the available treatment options. Additionally, patients with advanced stage of CRC are normally treated with chemotherapeutic drug, 5-fluorouracil (5-FU) alone or in combination of adjuvant such as oxaliplatin and avastin [3, 5–7]. Although these treatment regimens are effective at improving disease and overall survival (OS), severe side-effects such as severe nausea, vomiting, weight loss, and risk of infectious complications due to immunosuppression often burden the patients.

Even though both diseases are complex and multifarious by nature, both CRC and diabetes mellitus share various similar clinical risk factors which include age, diet, obesity, and gender [8, 9]. Furthermore, in the last decade, the pathogenesis and pathophysiological mechanisms of both CRC and type 2 diabetes mellitus (T2DM) related conditions such as hyperglycaemia, hyperinsulinemia, and insulin resistance are found to be closely related since they both involve the regulation of the insulin/insulin-like growth factor (IGF) signalling pathway [10, 11]. For instance, hyperinsulinemia and insulin resistance are found to promote the progression of tumorigenesis via either the insulin receptor in the epithelial tissues or by modulating the levels of other modulators, such as insulin-like growth factors (IGFs), sex hormones, inflammatory processes, and adipokines. This is due to the relative insulin sensitivity of the epithelial cells that enhances insulin-mediated signalling that induces cancer cell proliferation and metastasis [10, 11].

Metformin (1,1-dimethylbiguanide), a product of French lilac (*Galega officinalis*), is an oral biguanide and hypoglycemic agent that is prescribed to over 120 million patients with gestational diabetes [12, 13], T2DM [14, 15], non-alcoholic fatty liver disease [16, 17], premature puberty, [18] and polycystic ovarian syndrome (PCOS) [19, 20] worldwide. Unlike other biguanides such as sulfonylurea, and thiazolidinediones, the oral consumption of metformin is beneficial since it reduces the risk of cardiovascular disease by reducing cholesterol levels as well as inflammatory and blood clotting markers while controlling the blood glucose level [21, 22]. The presence of two methyl substitutes in metformin reduces the lipophilicity of metformin that aides the hepatic lactate clearance and excretion of metformin unchanged in the urine as compared to other diabetic drugs. Furthermore, metformin has several advantages in treating T2DM and associated cancer risks as compared to exogenous insulin and insulin secretalogues such as sulfonylurea drugs, which are reported to increase cancer risk and recurrence [23, 24]. Since metformin primary actions significantly reduce the circulating glucose and plasma insulin, hence, it improves insulin resistance in peripheral tissue. Therefore, the repurposed use of metformin may be beneficial in reducing the risk of diabetes related cancer incident [25]. Moreover, numerous lines of empirical evidence have supported the use of metformin as an anticancer agent that inhibits the transformative and hyperproliferative processes with anti-angiogenesis, radio-chemosensitizer, and antimetabolic effects that suppress carcinogenesis [25–27]. For example, in glioma models, the use of metformin in combination with five other repurposed drugs (itraconazole, naproxen, pirfenidone, rifampin, and quetiapine; known as EMT inhibiting sextet (EIS)) is shown to inhibit glioblastoma cells proliferation, invasion, chemoresistance, and metastatic activities which further blocked the epithelial to mesenchymal transition (EMT) [28]. The anticancer property of metformin is largely attributed to its capability in modulating signaling pathways involved in cellular proliferation, apoptosis, and metabolism. For instance, metformin modulates the synergistic regulation between AMPK, GSK-3β, and PPAR-γ that confer its anti-angiogenic, anti-invasive, and anti-proliferative as observed in pancreatic cancer and glioblastoma multiforme (GBM) [29].

In the last decade, mounting evidence support the use of metformin in the prevention and treatment of CRC (reviewed in later sections). Furthermore, extensive in vitro and in vivo research activities have successfully elucidated the molecular mechanisms of metformin in CRC models (discussed in later section). Moreover, the use of metformin as monotherapy or as an adjuvant in CRC intervention has led to further dose reduction and increased radio-chemosensitivity which lead to minimal gastrointestinal side effects and reduced toxicity. Furthermore, since metformin is relatively cheaper than other chemotherapy drugs and adjuvants, it may serve as a cost-effective and well affordable treatment option for CRC intervention. Nonetheless, contradictory population-based studies as well as beneficial metformin use among non-diabetic cancer patients further rationalize the need to systemically evaluate its effectiveness against CRC. Currently, researchers are hoping to get the better prospect form management of CRC along with the treatment of diabetes. The review article also highlights the use of metformin with improved survival among CRC patients with T2DM as compared to sulfonylureas and insulin. The present review aims to provide comprehensive and up to date preclinical, clinical, and epidemiologic reports on metformin as well as its molecular mechanisms that justify its repurposed use as a prospective and potential medicament in the intervention of CRC worldwide.

## The preclinical evidence use of metformin in CRC

### Metformin in *in vitro* CRC models

A series of successful pre-clinical reports (summarized in Tables 1 and 2) of metformin on CRC studies has led to its use as a potential therapeutic in patients. Additionally, metformin-loaded solid lipid nanoparticles have been designed to potentiate its therapeutic value [30]. The initial anticancer effect of metformin in CRC model was reported by Zakikhani et al., (2008) [31] where metformin concentration-dependently (2.5–20 mM, 72 h) reduced the proliferation of HT-29 cells. Metformin (5–20 mM, 72 h) activates the AMPK (phospho-AMPKα; Thr172) that inhibits the HT-29 and PC-3 cell growth. AMPK activation is associated with S6K inactivation (Ser235/236) in both HT29 and PC-3 cells [31]. In another study, metformin (1–10 mmol/L) for 72 h suppresses SW-480 cells proliferation in both concentration- and time-dependent manner by arresting the G_0_/G_1_ phase [32]. In a different report, higher concentration of metformin (10, 25, and 50 mM) inhibits HT29 cell growth in concentration- and time-(24 and 48 h) dependent manner and induces cellular apoptosis and autophagy as evident by increased expression of APAF-1, caspase-3, PARP, and Map-LC3 [33]. Moreover, metformin promotes apoptotic and autophagic cell death by suppressing the activation of nuclear factor E2-related factor 2 (NRF-2) and NF-κB in HT29 cells. The combination of metformin (5 mM for 120 h) with 4-iodo-6-phenylpyrimidin (4-IPP, 100 μM for 24 h) synergistically promotes apoptotic cell death in two organoid models from peritoneal metastases of CRC patients [34]. While 4-IPP inhibits AMPK, Akt, and JNK signalling, the long term addition of metformin enhances the activation of AMPK that reduces anabolic factors ribosomal protein S6 and p4EBP-1 activities which promotes depolarization of mitochondrial respiratory chain complex I. In CaCo2 cells, metformin (5, 10, 20, 50, and 100 mM, 48 h) significantly decreased the cell viability (up to 96% reduction) [35] even at the lowest concentration of 5 mM. Moreover, metformin alters the methylation status of tumor suppressor gene Ras asscociation domain family 1 isoform A (RASSF1A) which induces apoptosis, cell cycle arrestment, and inhibits cell migration.

**Table 1:** The summary of preclinical (in vitro) use of metformin in CRC models

CRC model	Main findings	Ref.
HT29 cells	Concentration-dependent anti-proliferative of metformin (2.5–20 mM, 72 h) that inhibits HT-29 growth by activating the AMPK (phospho-AMPKα; Thr172). Metformin (10, 25, and 50 mM) inhibits cell growth in concentration- and time-(24 and 48 h) dependent manner by inducing apoptosis and autophagy (increased expression of APAF-1, caspase-3, PARP, and Map-LC3) through oxidative stress (inactivation NRF-2 and activation NF-κB in HT29 cells.	[31, 33]
SW620 cells	Metformin (1–10 mmol/L, 72 h) suppresses proliferation in both concentration- and time-dependent manner via arresting the G_0_/G_1_ phase. Metformin (5 mM, 2 h) induces apoptosis in hypoxic SW620 cells and enhanced with co-treatment of (E)-4-((2-(3-oxopop-1-enyl)phenoxy)methyl) pyridinium malonic acid Metformin in combination with 5-FU significantly enhances antiproliferative, apoptosis, and cell-cycle arrestment in SW620 cells.	[32, 37, 45]
Organoid models from peritoneal metastases of CRC patients	Combination of metformin (5 mM for 120 h) with 4-IPP (100 μM, 24 h) synergistically promotes apoptosis by activating AMPK that reduces ribosomal protein S6 and p4EBP-1 activity that depolarizes mitochondrial respiratory chain complex I.	[34]
CaCo2 cells	Metformin (5–100 mM, 48 h) significantly decreases cell viability (up to 96% reduction) and edits the methylation status of RASSF1A which causes cellular apoptosis, cell cycle arrestment, and cell migration.	[35]
Human LoVo and mouse MCA38 cells	Metformin (10 μg/mL) alone and in combination with adinopectin (20 μg/mL) for 24 h suppresses IL-1β-induced malignant potential via STAT3 and AMPK/LKB1 signaling pathways. Co-administration with IL-1β increases the Sub-G1 population and decreases the G1 and/or S population by modulating cyclin E2, p21, and p27 expression.	[36]
COLO 205 cells	Combination of metformin (10 mM) with silibinin (100 mM) demonstrates a better antiproliferative activity as compared to either metformin (20 mM) or silibinin (200 mM) alone without any cytotoxic effects on the normal HCoEpiC.	[38]
HCT116 cells	Low concentration (60 μM) in combination with genistein (2 μM) and lunasin (2 μM) increases PTEN expression, inhibits cancer stem cell-likecells CD133^+^CD44^+^ subpopulation, and reduces FASN expression. Metformin (5–20 mM) synergistically (with 5-FU and oxaliplatin); known as FuOx; 200 μM 5-FU and 5 μM oxaliplatin) induces cell death, inhibits colonospheres formation, enhances colonospheres disintegration, and suppresses CRC cell migration. FuOx combination inactivates Akt with increased miRNA145 (tumor suppressive) and reduction in miRNA 21 (oncogenic) expression. Additionally Wnt/β-catenin signaling pathway and transcriptional activity of TCF/LEF, β-catenin as well as c-myc expression were inhibited in HCT-116 cells. Metformin (5 mM) and 5-FU (25 μM) enhances antiproliferative and migration through the silencing of miR-21 expression that increases the Sprouty2. Metformin (1–10 mM, 24–48 h) induces clonogenic cell death in both wild-type p53 HCT-116 (HCT116 p53^+^/^+^) and p53-deficient HCT-116 cells (HCT116 p53^−^/^−^) and augments radio-sensitization towards IR in HCT116 p53^−^/^−^ cells. Metformin (10 mM) suppresses LCA (30 μM)-oxidative stress by inactivating NF-κB and downregulating IL-8. Metformin-treated conditioned media inhibits of HUVECendothelial cell proliferation and tube-like formation. . Metformin (1–4 mM, 24–72 h) reduces EMT in HCT116 sphere cells via inactivation of Wnt3α/β-catenin signaling (with reduction of Vimentin and increased epithelial marker). Consequently, metformin promotes sensitization of HCT116 sphere cells towards 5-FU treatment (25 μg/mL).	[39, 44, 47, 48, 50, 52]
Caco-2 and HCT116 cells	Addition of metformin to 5-ASA (48 h) inhibits the Caco-2 (13 mM of metformin and 2.5 mM of 5-ASA) and HCT-116 cells proliferation (13 mM of metformin and 2.5 mM of 5-ASA) and induces apoptosis by inducing oxidative stress and NF-κB inflammatory responses.	[40]
DLD-1, HT-29, Colo205 and HCT116	Metformin (2.5–10 mM) did not decrease the cell viability but sensitizes the cells towards TRAIL (50 ng/mL) that is followed with induction of extrinsic and intrinsic apoptosis through the suppression of Mcl-1 by promoting the dissociation of Noxa from Mcl-1 that activates E3 ligase Mule.	[41]
HT-29, SW620, and HCT116 cells	Metformin addition to sirolimus synergistically promotes the reduction cell viability (48 h) via downregulation of p-mTOR, p-70S6K, p-4EBP1, livin, survivin, E-cadherin, TGF-β, and pSmad3.	[42]
HT-29 and HCT116 cells	Single exposure (24 h) either 1,25D3 (10–1000 nM) or metformin (1–20 mM) reduces the cell viability in HCT116 (p53 wild-type), HCT116 (p53^−^/^−^), and HT-29 (p53 mutant). Both 1,25D3 and metformin synergistically promotes apoptosis, and autophagy irrespective of the p53 status in all of the cells tested via AMPK, intracellular ROS, Bcl-2, and increasing LC3II:LC3I ratio. Additionally, metformin addition in the combination treatment arrests cell cycle in G_2_/M phase (HCT116 p53^−^/^−^) and S phase (HT-29 cells).	[43]
	In a different report, metformin at 1 mM (24 h) increases the sensitization of HT29 cells to oxaliplatin (R = 2.66, P < 0.01) but no in HCT116 cells	[46]
DLD-1 cells	Metformin (5 mM, 24 h) synergistically promotes oxaliplatin (12.5 μM) cytotoxic and anti-proliferative b increasing HMGB1 expression via Akt and ERK1/2. Metformin activates AMPK signaling at lower concentration and short time exposure (0.5–2 μM, 1 h) prior to radiation leads to radioresistance.	[49, 54]
SW-480 and HT-29	Pretreatment with metformin (2 mM, 16 h) activates AMPK signaling that inhibits the phosphorylation of β-catenin and Akt (Ser473) induced by insulin (10 ng/mL)or IGF-1 (10 ng/mL).	[51]
HCT116, RKO and HT-29 cells	Metformin (1 and 5 mM, 24 h) did not inhibit the proliferation and daily treatment (5 mM, 2 weeks) did not suppress the anchorage-independent growth, apoptosis, autophagy, and cell cycle arrest.	[53]

**Table 2:** The summary of preclinical (in vivo) use of metformin in CRC models

CRC model	Main findings	Ref.
HT-29-xenografted BALB/c-nude mice	Co-administration of metformin (250 mg/kg) with sirolimus (1 mg/kg), tacrolimus (1 mg/kg) or cyclosporin A (5 mg/kg) for four weeks significantly suppresses the tumor growth in HT-29-xenografted BALB/c-nude mice by downregulating the expression of p-mTOR, p-70S6K, p-4EBP1, livin, survivin, E-cadherin, TGF-β, and pSmad3.	[42]
*Apc* mutated mice	Metformin (250 mg/kg/day, 10 weeks) reduces polyps number (2.0–2.5 mm) but increases polyps ranging from 1.0–1.5 mm in diameter in *Apc*^Min/+^ mice. No significant reduction in total number of polyps in the small intestine and changes in BrdU index, PCNA index, percentage of apoptotic cells, *cyclin D1* and *c-myc* as compared to untreated group. Metformin (250 mg/kg/day, 6–32 weeks) + basal diet inhibit formation of ACF in azoxymethane-induced mice. Treatment decreased total number of polyp formation (by 20%), polyp expansion (by 11%) and abolished polyps larger than 3 mm. Metformin suppressed the colonic epithelial cell proliferation (not by apoptosis) in the azoxymethane-induced mice.	[55, 56]
MC38-xenografts mice	Metformin mitigates high-energy diet-induced tumor growth in MC38-xenografts mice by reducing FASN expression.	[57]
Organoid peritoneal metastases of CRC patients xenografts	Metformin inhibits DMH-induced ACF formation in diabetic Sprague Dawley rats by reversing the Warburg effect.	[58]
COLO25 and DSS-mice	Metformin significantly suppressed TNF-α-stimulated COLO 205 cells and ameliorated DSS-induced acute colitis and colitic cancer in IL-10^−^/^−^ mice.	[59]
SW48-Mut xenograft nude mice	Pre-administration of metformin (one week) reduces tumor volume in a time-dependent manner (maximum inhibition ~ 50%) in SW48-Mut xenograft nude mice.	[60]
HCT116 and HT-29-xenograft SCID mice	FuOx mixture (metformin (5 weeks) + 5-FU (IP, 25 mg/kg, once a week for 3 weeks) and oxaliplatin (IP, 2 mg/kg, once a week for 3 weeks)) inhibited tumor volume (50%, day 34) in HCT116-xenografts and in HT-29-xenografts (more than 70%). FuOx downregulated *CD44*, upregulated *CK2*0, and reduced number of stem/stem like cells Metformin (IP, 250 mg/kg/day) prior to IR inhibits 59% tumor growth as compared to 4.5% in metformin-treated only and IR-treated only HCT116 p53^−^/^−^ xenografts mice. Combination with IR inhibits DNA repair protein that increases radiosensitivity in HCT116 p53^−^/^−^ xenografts mice. Metformin (alone, 150 mg/kg body weight) and with rapamycin (intraperitoneal, 0.5 mg/kg body weight) modulates AMPK and mTOR modulation, inhibits tumor volume in HCT116-xenorafted NOD/SCIDs male mice. The addition of probiotic mixture inhibited the intracellular ROS, IL-3, and IL-6 levels which further reduced the tumor volume by 40%.	[44, 48, 63]
DMH-induced CRC in diabetic and non-diabetic mice	Single (100 or 200 mg/kg) and combination of metformin and/or oxaliplatin inhibited angiogenesis and tumor proliferation in DMH-induced CRC diabetic and non-diabetic mice by suppressing tumor angiogenesis and cell proliferation by reducing serum VEGF level and intratumoral IGFR-I.	[61]
PDX- female SCID mice	Metformin (150 mg/kg, 24 days) suppresses tumor growth (by 50%) in PDX CRC-female SCID mice. Combination with 5-FU (IP, 25 mg/kg) inhibited tumor growth (up to 85%). Metformin exposure to ex vivo PDX organoids culture suppresses O_2_ via activation of AMPK signaling and inhibited culture growth.	[62]
DMH-induced CRC rat and DMH-DSS-induced colitis-associated colon neoplasia mice model	Metformin (medium dose of 120 mg/kg/day) + vitamin D3 (100 IU/kg/day) synergistically enhances the chemopreventive effects against DMH-induced colon cancer rat and DMH-DSS-induced colitis-associated colon neoplasia mice model	[64]

Metformin administration alone (10 μg/mL) and in combination with adinopectin (20 μg/mL) for 24 h, suppress IL-1β-induced malignant potential in human (LoVo) and mouse (MCA38) colon cancer cells via STAT3 and AMPK/LKB1 signaling pathways [36]. Furthermore, co-administration of metformin with IL-1β increases the Sub-G_1_ population and decreases the G_1_ and/or S phase population by modulating cyclin E2, p21, and p27 expression. Additionally, the combination of adinopectin and metformin, co-administered with IL-1β further enhances the anticancer effects of metformin. Metformin (5 mM for 2 h) also induces apoptosis in hypoxic SW620 cells which is further enhanced following co-treatment with cinnamaldehyde derivative, (E)-4-((2-(3-oxopop-1-enyl)phenoxy)methyl) pyridinium malonic acid [37]. Combination of metformin (10 mM) with silibinin (100 mM) demonstrates a better antiproliferative activity in COLO 205 cells as compared to either metformin (20 mM) or silibinin (200 mM) alone without any cytotoxic effects on the normal colon cells, HCoEpiC [38]. In another report, low concentration of metformin (60 μM) in combination with genistein (2 μM) and lunasin (2 μM), increased the PTEN expression, inhibited the cancer stem cell-like cells CD133^+^CD44^+^ subpopulation, and reduced fatty acid synthase (FASN) expression in HCT116 cells [39]. These observations were followed by inhibition of colonosphere formation and cell proliferation. The addition of metformin to 5-aminosalicylic acid (5-ASA) for 48 h significantly inhibits the Caco-2 (13 mM of metformin and 2.5 mM of 5-ASA) and HCT-116 cells proliferation (13 mM of metformin and 2.5 mM of 5-ASA) and induces apoptotic cell death via modulation of oxidative stress and NF-κB inflammatory responses [40]. Although the exposure to metformin (2.5–10 mM) in human CRC cells (DLD-1, HT29, Colo205 and HCT116) did not decrease the cell viability to 50%, its exposure (10 mM) sensitized the cells towards TRAIL (50 ng/mL) [41]. This sensitization effect was followed with extrinsic and intrinsic apoptosis through the suppression of myeloid cell leukemia 1 (Mcl-1). Although metformin addition did not influence the *Mcl-1*, it significantly enhanced the Mcl-1 protein degradation and polyubiquitination by promoting the dissociation of Noxa from Mcl-1 that activated E3 ligase Mule. Additionally, metformin is also reported to enhance the anticancer effects of immunosuppressants in vitro and in vivo CRC models [42]. Metformin addition into sirolimus synergistically promotes the reduction of HT29, SW620, and HCT116 cell viability. In HT29 xenografted BALB/c-nude mice, the daily combination administration of metformin (250 mg/kg) with sirolimus (1 mg/kg), tacrolimus (1 mg/kg) or cyclosporin A (5 mg/kg) for 4 weeks significantly suppresses the tumor growth. Further mechanistic study reveals that combination of metformin and sirolimus downregulates the expression of p-mTOR, p-70S6K, p-4EBP1, livin, survivin, E-cadherin, transforming growth factor (TGF-β), and pSmad3 protein expression in both in vitro and in vivo experiment. In different p53 status CRC cell lines, the single exposure (24 h) to either 1,25D3 (10, 50, 100, 500, and 1000 nM) or metformin (1, 2, 5, 7.5, 10, and 20 mM) reduces the cell viability in HCT116 (p53 wild-type), HCT116 (p53^−^/^−^), and HT-29 (p53 mutant) [43]. However, both 1,25D3 and metformin demonstrate the most pronounced effect in wild type 53 HCT116 cells. The combination of 1,25D3 (100 nM) and metformin (increasing concentration) results in synergistic effects, apoptosis, and autophagy irrespective of the p53 status in all of the cells tested. Nevertheless, the combination effect induces AMPK, intracellular ROS, Bcl-2, and increases LC3II:LC3I ratio which is more pronounced in the wild type p53 cells. Additionally, metformin in the combination treatment regime is responsible for arrestment of cell cycle in G_2_/M phase (HCT116 p53^−^/^−^) and S phase (HT-29 cells). These observations suggest that although p53 status does not affect the synergistic anti-proliferative activity of metformin and 1,25D3, it influences the molecular signaling and cellular responses of the CRC models.

Nangia-Makker et al. [44] demonstrated that metformin (5–20 mM) synergistically in combination with 5-fluorouracil (5-FU) and oxaliplatin (FuOx; 200 μM 5-FU and 5 μM oxaliplatin) induced cell death in HT-29 and HCT-116 cells. The combination treatment (1.25–10 mM of metformin, 50 μM of 5-FU and 1.25 μM of oxaliplatin) significantly inhibited colonospheres formation, enhanced colonospheres disintegration, and suppressed the cell migration by 7–8 folds as compared to untreated cells. The combination of metformin and FuOx inactivated the Akt with increased miRNA 145 (tumor suppressive) and dereased in miRNA 21 (oncogenic) expression. Additionally, the combination treatment inactivated the Wnt/β-catenin signaling pathway and inhibited the transcriptional activity of TCF/LEF, decreased total β-catenin as well as c-myc expression in HCT-116 cells. Zhang et al. [45] demonstrated metformin in combination with 5-FU significantly synergized the apoptosis and cell-cycle arrestment in SW620 cells. In a different report, metformin at 1 mM (24 h) increases the sensitization of HT29 cells to oxaliplatin (R = 2.66, *P* < 0.01) but not in HCT116 cells [46]. Feng et al. [47] demonstrates that the suppression of HCT-116 cells proliferation and migration by metformin (5 mM) and 5-FU (25 μM) can be potentiated by knocking down miR-21 expression which in turn increases the Sprouty2, a tumor suppressor gene expression. In a different study, metformin (1–10 mM, 24–48 h) induces clonogenic cell death in both wild-type p53 HCT-116 (HCT116 p53^+^/^+^) and p53-deficient HCT-116 cells (HCT116 p53^−^/^−^) [48]. Moreover, metformin augments the radio-sensitization towards ionizing radiation (IR) in the HCT116 p53^−^/^−^ cells as compared to the wild-type group by suppressing the DNA repair protein expression and prolonging the cell cycle arrestment.

Other than enhancing the effect of chemotherapeutic drugs, metformin also potentiates the adjuvant activity in CRC models. Metformin (5 mM, 24 h) synergistically promotes oxaliplatin (12.5 μM) cytotoxic and anti-proliferative effects in DLD-1 cells [49]. The single treatment with oxaliplatin (2.5–25 μM, 1–24 h) in DLD-1 cells promotes the expression of high-mobility group box 1 protein (HMGB1) via Akt and ERK1/2 that induces chemoresistant against chemotherapeutic drugs. Interestingly, metformin reverses this observation by reducing the HMGB1 expression that promotes the cytotoxic effect of oxaliplatin in DLD-1 cells. The findings from this study suggest the incorporation of metformin in current CRC adjuvant setting that can reduce the chemoresistant and enhance the cytotoxicity against CRC tumor. Carcinogenesis through angiogenesis can be associated with promotion of inflammation by the augmentation of intracellular ROS. Metformin addition (10 mM) significantly suppresses lithocholic acid (LCA, 30 μM)-induced intracellular ROS level in HCT116 cells [50] via the inhibition of NADPH oxidase that consequently inactivates NF-κB and concomitantly downregulates IL-8. Moreover, the metformin-treated conditioned media inhibits HUVEC endothelial cell proliferation and tube-like formation as compared to LCA-treated conditioned media, suggesting metformin anti-angiogenic activity. As previously discussed, hyperinsulinemia can lead to insulin-mediated signalling and insulin resistance that promotes CRC progression and metastasis. However, pretreatment with metformin (2 mM, 16 h) in SW-480 and HT-29 activates AMPK signaling that inhibits the phosphorylation of β-catenin and Akt (Ser473) induced by insulin (10 ng/mL) or IGF-1 (10 ng/mL) [51]. In a study setting, metformin modulates the stemness of CRC cells by reducing the epithelial–mesenchymal transition (EMT) as observed in HCT116 sphere cells [52]. The cells exposure to metformin (1–4 mM, 24–72 h) results in the inactivation of the Wnt3α/β-catenin signaling that leads to reduction of mesenchymal marker Vimentin and increased epithelial marker which further decreases HCT116 sphere cells resistant towards 5-FU treatment (25 μg/mL), highlighting metformin capability of suppressing CRC EMT transition while promoting sensitization towards 5-FU.

Despite the monumental encouraging reports, another study demonstrated that the administration of metformin (1 and 5 mM for 24 h) did not significantly inhibit the proliferation of HCT116, RKO and HT29, CRC cells. Metformin daily treatment (5 mM) for 2 weeks did not suppress the anchorage-independent growth in all of the cells. Moreover, Sui et al. [53] reported that metformin treatment (1, 5 and 10 mM) for 24 h did not induce anchorage-independent growth, apoptosis, autophagy, and cell cycle arrest in HCT116, RKO and HT29 cells, which suggests that metformin do not possess antineoplastic activity when used as a single agent, contradictory to other findings. These contradictory findings can be due to the different concentration and time exposure applied in the experiment setting. The use of 1–5 mM with a shorter time frame of 24 h as compared to 5–20 mM for 24–72 h in most of the in vitro studies could suggest that metformin induces its anticancer effects in CRC cells at higher concentration with longer time incubation. In another contradictory report, the activation of AMPK signaling by metformin at lower concentration and short time exposure (0.5, 1 and 2 μM, 1 h) prior to radiation leads to radioresistance in DLD-1 cells [54]. When the cells were knockdown with AMPK siRNA or treated with compound C, the DLD-1 cells were resensitized towards the radiation. Although the report contradicts other findings, it is important to note that the pretreatment with metformin at lower dose (below 2 μM) at a shorter time duration may be responsible for this contradictory observations.

### Metformin in *in vivo* CRC models

The increased risk of cancer among diabetic patient is postulated to be associated with the hyperglycemic characteristic of the cancer cells that require high glucose usage to compensate the high metabolic activity. Therefore, various in vivo studies have investigated the beneficial use of metformin as antidiabetic and anticancer agent in CRC. The use of metformin as an anticancer agent against CRC can be associated with the inhibition of polyps growth in the intestine. In *Apc* mutated mice, metformin treatment (250 mg/kg/day for 10 weeks) significantly decreases the number of polyps ranging 2.0–2.5 mm in diameter but increases the number of polyps ranging 1.0–1.5 mm in diameter in *Apc*^Min/+^ mice [55]. Moreover, the analysis of BrdU index, PCNA index, percentage of apoptotic cells, and gene expression of *cyclin D1* and *c-myc* in tumor tissues of metformin-treated group demonstrates no significant alteration as compared to untreated group. The authors reported that metformin treatment did not significantly reduce the total number of polyps in the small intestine as compared to the untreated groups (42.11 ± 4.76 vs 38.22 ± 4.53; number of polyp/mouse, respectively). These observations suggest that metformin inhibits the intestinal polyps growth by reducing their size but not by inhibiting the total number of intestinal polyps, tumour cell proliferation or activation of apoptosis. In a follow up study, treatment with metformin (250 mg/kg/day) and basal diet combination for 6–32 weeks significantly inhibits the development of aberrant crypt foci (ACF) per mouse by 68.5 and 58.6%, respectively against azoxymethane (AZM)-induced mice [56]. Metformin treatment for 32 weeks also modestly suppressed the total number of polyp formation (20% reduction) and polyp expansion (11% size reduction) where the appearances of polyps that are larger than 3 mm were abolished in the metformin-treated mice. Additionally, metformin decreased the BrdU and PCNA indices but did not induce apoptosis in the AZM-induced mice, which indicates that metformin suppresses the ACF formation by suppressing the colonic epithelial cell proliferation.

Algire et al., (2010) [57] first demonstrated that metformin possessed the ability to mitigate the effect of high-energy diet in promoting the growth of tumors in MC38-xenografted mice. The addition of metformin significantly reduced diet induced hyperinsulinemia and FASN which reduced tumor growth and volume. Additionally, metformin also inhibits DMH-induced formation of colorectal aberrant crypt foci (ACF) in diabetic Sprague Dawley rats by reversing the Warburg effect [58]. Metformin is also beneficial in treating inflammatory bowel disease (IBD) and the chronic or long-term IBD can induce the development of colitis-associated colon cancer (CAC). Koh et al., (2014) [59] demonstrated that metformin significantly suppressed TNF-α-stimulated COLO 205 cells and ameliorated dextran sulfate sodium (DSS)-induced acute colitis and colitic cancer in IL-10^−^/^−^ mice. Additionally, in the dietary restriction (DR)-resistant tumors model, 1 week pre-administration of metformin time-dependently reduces the tumor volume (maximum inhibition of approximately 50%) in SW48-Mut xenograft nude mice [60].

In recurrence CRC model, the regimen treatment of metformin (5 weeks) in combination with mixture of 5-fluorouracil (IP, 25 mg/kg, once a week for 3 weeks) and oxaliplatin (IP, 2 mg/kg, once a week for 3 weeks) (mixture known as FuOx) demonstrated positive inhibitory effects of CRC in SCID mice [44]. Metformin in combination FuOx suppressed the tumor volume (by almost 50%) by day 34 post-injection in HCT116-xenograft mice and rapidly inhibited the tumor volume by more than 70% in HT-29-xenograft mice. These observations were associated with the downregulation of *CD44*, upregulation of *CK2*0 gene expression, and reduced number of stem/stem like cells. In another study, Zaafar et al. [61] demonstrated the single and combination of metformin and/or oxaliplatin metformin that inhibited DMH-induced colon cancer in diabetic and non-diabetic mice by suppressing tumor angiogenesis and cell proliferation. Metformin treatment (100 or 200 mg/kg) reduced the serum VEGF level and mitigated the intratumoral cell proliferation with greater efficacy in the diabetic than the non-diabetic mice. The combination treatment of oxaliplatin and metformin significantly led to a greater reduction in serum VEGF level with decreased intratumoral IGFR-I and intra-tumoral vascular density. In a different model, daily oral administration of metformin (150 mg/kg, 24 days) suppresses the tumor growth by 50% in the patient-derived xenograft (PDX) lines from two CRC patient on female SCID mice [62]. Interestingly, when combined with 5-fluorouracil (IP, 25 mg/kg), the tumor growth was further inhibited up to 85%. Additionally, metformin exposure to ex vivo culture of organoids generated from PDX models modulated the metabolic changes and inhibited the culture growth by suppressing the O_2_ consumption through activation of AMPK signaling. Although the anticancer effect of metformin was shown to be largely attribute to AMPK and mTOR modulation, its oral administration (150 mg/kg body weight) alone or in combination with rapamycin (intraperitoneal, 0.5 mg/kg body weight) only resulted in 20% of tumor volume inhibition in HCT116-xenografted NOD/SCIDs male mice [63]. However, addition of probiotic mixture (*Lactobacillus rhamnosus, Saccharomyces boulardii, Bifidobacterium breve, Bifidobacterium lactis, Lactobacillus acidophilus, Lactobacillus plantarum* and *Lactobacillus reuteri*) inhibited the intracellular ROS, IL-3, and IL-6 levels which further reduced the tumor volume by 40%.

Other than potentiating the chemosensitivity of chemotherapeutic drugs, the addition of metformin (IP, 250 mg/kg, once a day) prior to ionizing radiation (IR) demonstrates a better mitigation of the tumor growth up to 59% inhibition as compared to 4.5% in metformin-treated and IR-treated HCT116 p53^−^/^−^ xenografts mice [48]. Moreover, metformin addition into IR treatment delays the DNA repair through inhibition of DNA repair protein that leads to an increased radiosensitivity in HCT116 p53^−^/^−^ xenografts mice model. The combination of metformin (medium dose of 120 mg/kg/day) with vitamin D3 (100 IU/kg/day) enhances the chemopreventive effects against DMH-induced colon cancer rat and DMH-dextran sodium sulfate (DSS)-induced colitis-associated colon neoplasia mice models [64]. Medium dose of metformin and vitamin D3 demonstrated higher suppression in the total numbers of tumors (reduction of 67%), aberrant crypts (reduction of 51%) and total ACF (reduction of 49%) in DMH-induced colon cancer rats at 18 weeks. In addition, the combination of metformin and vitamin D3 further enhanced the inhibition of tumour numbers (more than 50%), tumour volume (up to70%) and incidence of noninvasive adenocarcinoma (100%) as compared to either metformin or vitamin D3 alone in DMN + DSS-induced colitis-associated colon neoplasia mice model. Contradictorily to all of the positive findings, metformin did not decrease the tumor size in HT-29-xenografted mice as compared to 5-Aminoimidazole-4-carboxamide ribonucleotide (AICAR), an AMPK activator [53]. The tumor size from the HT-29 xenografts of AICAR group, and not metformin group was smaller compared to control group.

## The clinical use of metformin in CRC intervention

In recent years, numerous empirical clinical evidence reported that metformin intervention may prevent and reduce the risk CRC at various stages [65–71]. In a case-control study, Sehdev et al. [72] reported a 12% risk reduction of CRC in the diabetic patients in USA following the use of metformin over a period of 12 months. Furthermore, a large number of meta-analysis comprises of case control and cohort studies demonstrate statistically significant reduction developing CRC in individuals who were taking metformin compared with non-receiving metformin with mild to moderate heterogeneity [73–75]. In two companion case–control studies conducted in Milan and Pordenone/Udine (Italy) and Barcelona (Spain) between 2007 and 2013, the prevalence of CRC was positively associated with diabetes. Moreover, the use of metformin was associated with a reduced CRC risk (odd ratio, OR 0.47, 95% and confidence interval, CI 0.24–0.92) risk as compared to increased CRC risk by insulin (OR 2.20, 95% CI 1.12–4.33) [76]. Furthermore, the study found that the long term use of metformin and insulin (over 10 years) either further reduces or strengthens the CRC risk with OR value of 0.36 and 8.18, respectively. The observation demonstrates the safer and beneficial use of metformin than insulin in reducing CRC risk among T2DM patients. Cardel et al., summarizes that from the total of 13 meta-analysis, 12 observational and 1 randomized studies that assessed the association between metformin and CRC, the risk of CRC is decreased by 17% (OR 0.83, 95% CI 0.74–0.92) among patients treated with metformin compared to those of not using metformin [77]. In another meta-analysis report that includes eight cohort studies and three case-control studies, metformin is associated with 25% reduction of CRC incidence among T2DM patients [78]. A meta-analysis study reveals that metformin therapy decreased the risk of all cause of fatality by 44% and the risk of CRC specific fatality by 34% in diabetic CRC patients with an improvement in the overall survival (OS) as compared to those in non-metformin patients [79]. In a more recent analysis (12 cohort studies, 7 case-control studies and 1 randomized controlled trial study), metformin intake is associated with 25% reduction of colorectal adenoma incidence (OR 0.75, 95% CI 0.59–0.97) and 22% decreased of CRC risk (OR 0.78, 95% CI 0.70–0.87) in T2DM metformin users than T2DM non-metformin patients [80]. Metformin is also thought to be beneficial in preventing the incidence of CRC among diabetic patients with previous history of CRC either in T2DM or non-diabetic patients. In a recent systemic review and meta-analysis consisting ten studies (8726 patients), it is concluded that metformin use decreases the risk of adenoma (OR = 0.76, 95% CI 0.63–0.92) especially in high-risk population (patients with colorectal neoplasia history, OR = 0.61, 95% CI 0.34–1.10) and in high risk population with T2DM (OR = 0.75, 95% CI 0.62–0.91) [81]. In another report encompasses 11 studies, although metformin intake did not protect against the risk of the total adenoma (OR = 0.86, *p* = 0.274) and adenoma recurrence (OR = 0.89, *p* = 0.137), its intake significantly reduced the risk advanced adenoma (OR = 0.51, *p* < 0.001) [82].

In an epidemiological study, metformin reduced the risk and incidence of colorectal adenomas (median follow-up of 58 months) among consecutive diabetic patients with CRC history in Seoul, Korea. The study found that only 33 patients (28.9%) exhibited adenomatous colorectal polyps among the 114 metformin user as compared to 58 (46.0%) patients who developed colorectal adenomas among 126 patients non-metformin user [83]. Zhang et al. [45] reported that metformin use in 86 CRC patients with T2DM significantly reduced the proportion of patients with poorly differentiated adenocarcinoma (2.78% vs 16.0%) and distant metastasis rate (5.60% vs 21.6%) than the non-metformin group in Guangzhou, China. Fransgaard et al. reported that, metformin intake improved the OS among 1962 diabetic CRC patients who underwent surgery and reduced the mortality rate by 15% as compared to patients who were treated with insulin [84]. In a surveillance epidemiology and endpoint research-medicare database study, the combination use of metformin with DPP4 inhibitors further promoted the survival advantage of CRC patients with hazard ratio (HR) of 0.83 and CI of 0.77–0.90 (*P* < 0.0001) as compared to the use DPP4 inhibitors alone (HR:0.89; CI: 0.82–0.97, *P* = 0.007) [85]. The use of DPP4 inhibitors alone further demonstrated positive trend of survival advantage in CRC patients, although it did reach significant statistic threshold with HR value of 0.87 and CI value of 0.75–1.00 (*P* = 0.055). Likewise, the combination administration of metformin and DPP4 inhibitors resulted in a higher and significant survival advantage with HR value of 0.77 and CI value 0.67–0.89 (*P* = 0.003). Nevertheless, the encouraging data from this epidemiology study would need to be further strengthened with a bigger sample size.

The ability of metformin to reduce the CRC incident could be attributed to its capability to intervene the development of colorectal polyps and adenomas either in T2DM or non-diabetic patients as reported in some clinical studies [86–88]. For instance, in a prospective, randomized, placebo clinical trial, metformin intake decreased the mean number of aberrant cryptic foci in nondiabetic patients after 30 days of treatment with metformin compared to placebo group of patients [86]. In a phase-3, double-blind, 1-year randomized, placebo controlled trial, the safety and chemopreventive effects of metformin (250 mg daily) on sporadic CRC (adenoma and polyp recurrence) in non-diabetic patients with a high risk of adenoma recurrence were assessed [87]. Colonoscopy examination shows that metformin intake (among 71 patients of metformin group) for 1 year was safe and effective in reducing the occurrence of total polyps (hyperplastic polyps plus adenomas) to 38% (27 out of 71 patients, 95% CI 26.7–49.3%) and of adenomas to 30.6% (22 out of 71 patients, 95% CI 19.9–41.2%) without serious side-effects as compared to the patients [62] who received placebo treatment (56.5 and 51.6%, respectively). The data is interesting since metformin is shown to be beneficial in reducing the prevalence of metachronous adenomas or polyps among non-diabetic patients as compared to most reports among T2DM patients. However, a larger sample and longer-term clinical trials are further required as to ascertain the capability of low dose of metformin in decreasing the total prevalence metachronous adenomas or polyps after polypectomy among non-diabetic CRC patients.

In a single-center retrospective study, Cho et al. [88] analyzed a total of 3105 T2DM patients (912 patients exposed to metformin and 2193 to non-metformin) that had colonoscopy between May 2001 and March 2013. Cho et al. [88, 89] observed that patients exposed with metformin displayed lower detection rate of colorectal polyp and colorectal adenoma as compared to non-metformin group. Furthermore, the use of metformin also resulted in the lower detection of advanced adenomas indicating that metformin reduced the incidence of adenomas that may transform into CRC and thus, is beneficial in preventing colon cancer in patients with T2DM. Kim et al. [90] retrospective study shows that metformin use in diabetic patients without previous CRC history independently reduced the incidence of advanced colorectal adenomas and follow-up study revealed that metformin decreased advanced adenomas development rate as compared to non-metformin group. The retrospective study by Kowall et al. [91] supports this observation among 4769 patients in Germany and United Kingdom. In a single-centre, single-arm phase 2 trial among 50 patients with refractory metastatic CRC, the combination of metformin (850 mg/day, oral) and 5-FU (425 mg/m^2^) exhibited in median progression free survival of 1.8 months and overall survival of 7.9 months [92]. Moreover, the treatment paradigm resulted in 22% of patients [11] that acquired tumour stabilization after 8 weeks (primary end point) that lasted with median progression free survival of 5.6 months and overall survival of 16.2 months. Another population retrospective study among veterans in USA reported that although CRC patients with diabetes exhibited lower overall survival as compared to non-diabetic patients, metformin use improved the overall survival by 13% as compared to the use of other anti-diabetic drugs [93]. In a different retrospective cohort study in Southeastern Ontario, Canada, diabetic CRC patients that took metformin exhibited a positive association with prognosis with significant longer OS (91% at 1 year, 80.5% at 2 years and 72.2% at 3 years) as compared to patients taking other than metformin (80.6% at 1 year, 67.4% at 2 years and 53.5% at 3 years) and non-diabetic patients (86.5% at 1 year, 77.7% at 2 years and 64.2% at 3 years) [94]. In a retrospective study involving 339 patients (including T), a decremental trend was observed for adenoma detection rate in groups receiving insulin only, metformin only and insulin and metformin combination (40.9, 33.2 and 32.5%, respectively) although the *p* value is above 0.05 (*p* = 0.413) [95]. Similarly, the same trend was observed for advanced adenoma detection rate (18.2, 15.2 and 10.0%, *p* = 0.489). Although the reduction rates were not statistically significant, it is noteworthy that the intake of metformin and metformin in combination with insulin resulted in lower detection rates of adenoma and advanced adenoma among the subjects.

The intake of metformin is also associated with better CRC tumor response towards radiotherapy, especially among diabetic patients treated with neoadjuvant chemoradiotherapy in Korea [96]. In this study, T2DM metformin patients (*n* = 42) demonstrated significantly higher N downstaging (*p* = 0.006) and tumor regression grade 3–4 (*p* = 0.029) as compared to T2DM non-metformin (*n* = 29) and non-diabetic (*n* = 472) patients. However, the intake of metformin did not significantly affect the recurrence-free survival, disease-free survival, and OS rates which further suggest the use of metformin as neoadjuvant for chemotherapy in CRC patients. In a different report, metformin intake significantly improved prognosis among 202 veterans T2DM CRC patients in Tennessee, USA [97]. CRC patients with metformin intake recorded reduced death percentage (48% versus 76%, *P* < 0.001), recurrence rate (4% versus 19%, *P* = 0.002), metastases rate (23% versus 46%, *P* = 0.001), improved 5-year survival rates (57% versus 37%, *P* = 0.004), OS years (5.7 versus 4.1, *P* = 0.007), and enhanced reduction of carcinoembryonic antigen (72% versus 47%, *P* = 0.015) as compared to non-metformin CRC patients. In a population-based cohort study in Taiwan, Tseng, C. H [98]. reported that the longer duration use of metformin (≥ 3 years) in patients showed a significantly lower risk (27%) of CRC as well as chronic obstructive pulmonary disease (COPD) when compared to shorter exposure (< 1 and 1–3 years). In Ireland, the use of metformin among adult patients (207) with stage I - III CRC diagnosed from 2001 to 2006 showed a nonsignificant reduction in CRC–specific mortality in metformin-exposed patients as compared to non-metformin [108] and nondiabetic patients (3501) group based on the hazard ratios (HR). Nevertheless, the high-intensity use of metformin significantly demonstrated a reduction in CRC–specific mortality as compared to low-intensity metformin or metformin in combination with other antidiabetic drugs were studied and compared with other antidiabetic drugs only [99]. The high intensity or longer exposure to metformin was also shown to be beneficial in other population groups. In a population-based case-control study among the Danish citizens diagnosed with T2DM, the long term use of metformin (2000 mg within 5 years) only protected and reduced the risk of CRC among women than men [77]. This is by far the only gender specific report on metformin effect on CRC risk in T2DM patients, and thus, a bigger sample population and studies are required to validate this observation. Additionally, Cardel at al [77]. reported that the intake of metformin dose-dependently and time-dependently (> 250 defined daily dose (DDD) and for the duration > 1 year) decreased CRC risk.

Metformin has also been used as potential curative agent in combination with radiotherapy and/or chemotherapy regiment in a number of CRC intervention trials. Hyperinsulinemia and high level of IGF-1 are associated with CRC progression, thus, insulin based treatment among diabetic patients might impose the risk of CRC occurrence. Nevertheless, a retrospective study reported that combination of metformin and insulin lower the detection rate of colon adenoma (Ad) and advanced adenoma (Aad) to 32.5 and 10%, respectively. The Ad and Aad rates were lower when compared to insulin alone (Ad, 40.9% and Aad, 18.2%) and metformin alone (Ad, 33.2% and Aad, 15.2%), which suggested combination of metformin and insulin is more effective in reducing CRC risk among the T2DM patients [100]. In an observational study, metformin reduced CRC risk and improved the OS of patient group diagnosed with stage IV CRC who underwent curative resection [101]. However, metformin did not show any significant tumor response, change in target lesion size, progression free survival (PFS) rate, and OS rate in the palliative chemotherapy group. Additionally, metformin improved the tumor response to neoadjuvant concurrent chemoradiotherapy (CCRT) in locally advanced T2DM CRC patients [96]. The administration of metformin (250–800 mg/3x/day in T2DM patients with metformin history) in combination with neoadjuvant radiotherapy either chemotherapy regimen of intravenous 5-FU (425 mg/m^2^/day) and leucovorin (20 mg/m2/day) for 5 days during the first and fifth weeks) or a capecitabine-based (oral capecitabine (825 mg/m2/day) twice daily) was further validated in this study. Metformin use among 42 patients with T2DM demonstrated higher N and TRG 3–4 downstaging percentages (85.7 and 61.9%, respectively) as compared to non-metformin patients (51.7 and 34.5%, respectively). Nonetheless, there were insignificant different in OS and disease free survival (DFS) among the metformin, non-metformin, and non-diabetic patients. Currently, an ongoing randomized, phase II, double-blind, placebo-controlled trial aims to determine the effect of low-dose aspirin and metformin in stage I-III CRC patients since the single use of both drugs exhibited beneficial use in reducing adenoma recurrence and CRC mortality rates [102]. The CRC patients (*n* = 160) are divided in four arms; aspirin (100 mg/day), metformin (850 mg/bis in die), aspirin and metformin combination, or placebo for a duration of 12 months. This ASAMET trial aims to determine of the occurrence of adenoma (low, intermediate and/or high grade intraepithelial neoplasia) and prevalence of CRC recurrence at baseline and 12 months after randomization among the patients. Furthermore, the study uses the expression of biomarkers such as NF-κB, pS6K, p53, β-catenin, PI3K, and circulating IL-6, CRP and VEGF as the secondary outcomes. The data to be collected from this study is expected to provide or allow for a better early diagnosis steps of CRC recurrence and potential use of aspirin and metformin synergistic combination with an improved understanding for CRC intervention.

Since obesity is closely related to the onset progression of T2DM, the metformin effects CRC risk among elevated BMI patients with colorectal adenoma was studied in a phase IIa clinical trial on Southern California [103]. Patients with BMI above 30 and history of colorectal adenoma within past 3 years (age between 35 and 80, including non-diabetic patients) were enrolled and given metformin at a dose of 1000 mg over 3 weeks (end of study at 12 weeks). They reported that although 4 months intake metformin is safe in a non-diabetic patients, their body weight and glucose level were not significantly different before the beginning and completion of study. Moreover, metformin did not decrease the pS6 levels of biopsies rectal mucosa, although this protein is the main signalling target of LKB1/AMPK/mTOR in CRC models. This observation justifies the need to investigate metformin effects on the colorectum tissue itself to determine whether metformin may be pursued as an agent that might reduce CRC progression among non-diabetic and elevated BMI patients. Nevertheless, minimizing biases in of metformin intake in decreasing CRC risk remains pertinent among all clinical trials. A cohort study (encompasses 47, 351 diabetic patients without prior use of metformin) in Northern California between 1997 until 2012 was conducted to eliminate time-related biases factors (ever use, total duration, recency of use and cumulative dose among patients) [104]. They reported that no clear association between metformin ever use and CRC risk and no significant consistent trend of reduced CRC risk with increase metformin total duration, dose, or recency of use among the diabetic patients. Interestingly, the cumulative and long term use of metformin (more than 5 years) is found to reduce the CRC risk among the total population among current users (HR = 0.78, 95% CIs 0.59–1.04), particularly among the men diabetic patients (HR = 0.65, 95% CI 0.45–0.94). The similar trend was not observed among women diabetic patients, and this further warrant future studies that may explain on the observed gender bias on metformin effect in men. Additionally, patients that switched from sulfonylureas or added metformin intake in their current treatment also reported reduced CRC risk, which strengthen the beneficial use of metformin as T2DM and anticancer CRC agent. Additionally, although metformin use is safe among diabetic and non-diabetic patients, its intake does not affect the OS or PFS among diabetic patients of advanced (metastatic CRC) treated with first-line chemotherapy FOLFOX6 or FOLFIRI [105]. Moreover, the impact of metformin on non-diabetic CRC patients still remain vague. Currently, a number of completed and ongoing clinical trials (randomized, intervention) aim to determine on metformin efficacy in reducing CRC risk among non-diabetic, refractory as well as CRC cancer survivors [106–112]. Most of these studies are using DFS, OS, and PFS as the primary outcome measures between three to 5 year time frame following the intake of metformin (between 500 and 1000 mg/day/oral) in combination with chemotherapy agents, vitamin C, and exercises. Additionally, a MECORA study (phase 2 randomized clinical trial) is still ongoing with the aims to determine metformin impacts alone on the expression of biomarkers such as Ki67, cleaved caspase-3, and insulin resistance [112]. The results to be obtained from all of this clinical trial would be important in justifying the repurpose use of metformin as potential adjuvant CRC treatment and/or agent in reducing CRC risk among non-diabetic patients.

Although various clinical studies have reported the beneficial use of metformin in reducing the risk and protecting against CRC, in several studies, the observations are otherwise. In a report based on electronic database from Clinical Practice Research Datalink, UK, the use of metformin did not found to confer any benefit or protective effect against cancer, which include CRC among T2DM patients [113]. The record reveals out of 55,629 T2DM who were alive and cancer free at the entry of study, 2530 patients were diagnosed with cancer after a median follow-up of 2.9 years with HR ratio of 1.02 for all cancers. Nevertheless, the short median follow-up period may serve as a limitation factor in this report, thus, a longer follow-up period is required to further justify the protective impact of metformin on cancer, particularly CRC risk. In a randomized controlled trials (retrospective cohort studies), Tsilidis et al. [114] demonstrated the intake of initiators of metformin among T2DM patients within 12 months resulted in a similar total cases of cancer that includes CRC (HR 0.92; 95% CI 0.76–1.13) as compared to sulfonylurea group. Cossor et al. [115] conclude in their report the difference in OS against CRC among metformin use (*n* = 84), without metformin (*n* = 128), diabetes status (*n* = 1854 without diabetes) in postmenopausal women are not significant [105]. In a substudy of randomized TOSCA trial in Italy, metformin intake among T2DM CRC patients treated with fluoropyrimidine-oxaliplatin adjuvant chemotherapy neither associated with OS nor relapse free survival (adjusted HR, 1.51; CI, 0.48–4.77; *p* = 0.4781 and HR, 1.56; CI, 0.69–3.54; *p* = 0.2881, respectively) [116]. Among the T2DM CRC metformin users (76 patients, 63.3%), 26 patients demonstrated CRC relapsed (21.7%) and 16 patients died (13.3%) after a median follow-up of 60.4 months. However, since this a sub study of a randomized trial, a bigger population studies and a longer median follow-up are required to validate the impacts of metformin on CRC patients’ prognoses. In a different study (Mendelian randomization), the impacts of growth differentiation factor 15 (GDF-15), a potential biomarker for metformin intake was investigated on coronary heart disease, breast cancer, and CRC was evaluated [117]. Interestingly, GDF-15 is associated with reduced risk of coronary heart disease and breast cancer (OR: 0.93 and OR: 0.89, respectively) but not with T2DM and CRC risk factors. Garret et al., (2012) [118] reported in a retrospective study that metformin prolonged the OS of T2DM with CRC patients by 30% (improvement of 56.9 months to 76.9 months) as compared to other antidiabetic agents in USA. Additionally, a population-based cohort study among 1197 CRC patients between 1998 to 2009 in United Kingdom did not find any relevant or significant CRC protective evidence of metformin as well as other antidiabetic drugs use in CRC-specific death mortality before or after adjustment for potential confounders [119]. A nested case-control analysis in 920 diabetic patients (age 70 ± 8 years) in U.K. surprisingly revealed that the extensive intake of metformin (≥50 prescriptions) induced an insignificant increased risk of CRC in men [120], which is contradictory to the longer duration use of metformin discussed previously. It was also reported the use of metformin with adjuvant chemotherapy among stage III CRC patients with T2DM resulted in similar disease free survival, OS and time to recurrence with non-diabetic or T2DM patients without metformin [121].

Although these contradictory findings may not support the protective impacts of metformin against CRC, the incremental positive outcomes and report suggest otherwise. Collectively, these numerous positive observational studies and meta-analysis further strengthen the notion that metformin therapy is beneficial in reducing the risk and improving the survival of diabetic and non-diabetic CRC patients. All of these observations are summarized in Table 3.

**Table 3:** The summarization of metformin clinical use for CRC

Study population (Diabetic/Age/Gender/Stage)	Chemotherapy/Radiation/Surgery	Placebo/ Combined intervention/Drugs history	Dose & Duration of Treatment	HR /RR/Survival	Primary endpoint/secondary analysis	Life quality/Side-effects	Summary findings
Non-diabetic,151 patients 79 metformin, 72 placebo Randomized phase 3 trial single or multiple colorectal adenomas or polyps resected by endoscopy^87^	All had history of resection	i. Placebo controlled ii. Colonoscopies after one year treatment (71 metformin & 62 placebo)	250 mg/day for one year	i. Total polyps RR 0·67, 95% CI 0·47–0·97 ii. Adenomas RR 0·60, 95% CI 0·39–0·92	i. Total polyps Metformin vs Placebo 38% vs 56.5% ii. Total adenomas Metformin vs Placebo 30.6% vs 51.6%	11% side effects grade 1 No serious adverse effects	Low-dose metformin reduced metachronous adenomas or polyps after polypectomy
Diabetic and non-diabetic 50 patients with refractory metastatic CRC Age above 18 year-old (mean – 57 year-old)^92^	All were treated with chemotherapy and radiotherapy prior to study entry	5-fluorouracil (5-FU) Leucovorin	i. Metformin – 850 mg orally 2 times/day ii. 5-FU 425 mg/m^2^ iii. Leucovorin – 50 mg by I.V. Treatment were given for 28 days/cycle until patients death	Not mentioned	i. Disease control rate (DCR) at 8 weeks from staring of study ii. Progression-free survival (PFS), OS, and toxicity	Diarrhea, nausea, vomiting, and myelotoxicity	i. Treatment - median PFS of 1.8 months and OS of 7.9 months ii. 22% met primary end-point – median PFS of 5.6 months and OS of 16.2 months iii. Prolonged survival for obese and 5-FU off patients
482 patients (422 non-diabetic, 40 diabetic non-metformin, 20 diabetic metformin) with locally advanced rectal adenocarcinoma Age median is 58 to 63 year-old^70^	All were treated with chemotherapy, total mesorectal excision (TME) and radiotherapy prior to study entry	5-fluorouracil-based chemotherapy (98%) followed by adjuvant chemotherapy (81.3%) 15% insulin use in both diabetic merformin and non-metformin group	Not mentioned	Not mentioned	Pathologic complete response (pCR)	Not mentioned	i. Patients taking metformin had significantly increased disease-free (*P* = 0.013) and overall survival (*P* = 0.008) ii. Patients taking metformin had a significantly higher rate of pCR than either nondiabetics or diabetics non-metformin
86 diabetic CRC underwent resection 36 – metformin 50 – non user Age 29–88 years^45^	All had history of resection 37 – neither chemotherapy nor radiation history	5-fluorouracil (5-FU)	Not mentioned	Not mentioned	Metformin vs non-users i. incidence of metastasis 5.60% vs 21.6%, ii. CD133 expression 21.1% vs 50% iii. β-catenin expression 36.85 vs 72.2% iv. Poorly differentiated adenoma 2.78% vs 16.0%	Not mentioned	Metformin synergize 5-FU in reducing risk of poorly differentiated adenoma and metastasis incidence
240 diabetic without CRC history Age, metformin 58.8 ± 9.9 non user 61.6 ± 10.3^90^	No	i. Aspirin ii. NSAIDs iii. Sulfonylurea	Not mentioned	Metformin - Advanced adenoma RR 0.071	Colorectal adenocarcinoma – size, occurrence, number	Not mentioned	Metformin lower the risk of advanced CRC Adenomas in newly diagnosed patients
2088 cases (66–80 years) and 9060 control (61–77 years) January 2000 – December 2009^77^	YES	i. Aspirin ii. NSAIDs iii. Sulfonylurea	Cumulative 2000 g (DDD) within 5 years	i. Reduced risk of CRC OR 0.83, 95% CI 0.68–1.00 ii. Protective in women vs men (OR 0.66, 95% CI 0.49–0.90) vs (OR 0.96, 95% CI 0.75–1.23)	Not mentioned	Not mentioned	Dose and duration response - reduced risk of CRC metformin > 250 DDD and > 1 year Protective effect of long-term metformin against CRC in women
424 patients January 2004 – December 2008^108^	YES	i. Insulin ii. ADDs iii. Anti-cholesterol iv. Aspirin	Dose not mentioned, duration between 2005 and 2008	i. Metformin vs Non user − 76.9 vs 56.9 months (CI 61.4–102.4) vs CI 44.8–68.8) ii. 30% enhancement in overall survival (OS)	HbA1c level	Not mentioned	Metformin provided 30% improvement in OS as compared to other ADDs
2066 postmenopausal Women, 50–79 years 1854 non-diabetic 84 diabetic(+metformin) 128 diabetic(−metformin)^105^	YES	i. Insulin ii. Aspirin iii. NSAIDs	Median 4.1 years (3 days - 14.4 years)	i. Diabetic Metformin vs Non user - Non-significant (HR 0.78, 95% CI 0.38–1.55) ii. overall survival (HR 0.86, 95% CI 0.49–1.52)	i. Tumor size ii. Positive lymph nodes	Not mentioned	Non-significant difference specific survival in metformin compared to non-users
315 patients with stage I–III colorectal cancer from 2001 to 2006^77^	YES	i. 52% - Metformin + sulfonylurea ii. 72% -non metformin + sulfonylurea iii. Insulin iv. Other ADDs	Low and high intensity Duration - none	Low intensity HR 0.81, 95% CI 0.41–1.58 High intensity HR 0.44, 95% CI 0.20–0.95	Tumor grade/Size - Nonsignificant between metformin and non-users	Not mentioned	High-intensity metformin dosing reduced CRC-specific mortality
A) i. 856 patients with CRC from 2003 to 2005 ii. Age: men and women < 40 years B) i. 814 patients with CRC from 2003 to 2005 ii. Age: men and women > 40 years C). Diabetes status: ≥ 1 year and ≥ 3 years^76^	YES	None	i. < 1 year ii. 1–3 years iii. ≥ 3 years	< 40 years i. < 1 year - 0.876 ii. 1–3 years - 0.859 iii. ≥ 3 years - 0.643 > 40 years i. < 1 year - 0.896 ii. 1–3 years - 0.843 iii. ≥ 3 years - 0.646	none	Reduced incidence of COPD in metformin users compared to non-users	Significantly lower risk CRC by 27% Longer use of metformin inversely proportional to CRC
3775 underwent colonoscopy (May 2001 - March 2013) 912 with metformin 2193 non users Age > 40 years^88^	Colonoscopy	Not mentioned	i. < 1 year ii. 1–2 years iii. 2–3 years iv. ≥ 3 years	Not mentioned	i. Colorectal polyp - metformin vs non users (39.4% vs 62.4%) ii. adenoma - metformin vs non users (15.2% vs 20.5%) iii. Advanced adenoma - metformin vs non users (12.2% vs 22%)	Not mentioned	Metformin is beneficial in prevention of CRC
106 patients with stage IV CRC^83^	81chemotherapy 25 curative resection	Not mentioned	Not mentioned	Metformin improved free survival rate for curative group (HR0.024,95% CI0.001–0.435)	i. tumor response, ii. target lesion size Chemotherapy – non-significant	Lower recurrence incidence	Metformin reduced tumor recurrence after curative resection
8046 patient 2682 case group 5364 control group (60% male & 40% female each group) Age mean 55 and 57^72^	YES	i. statins ii. insulin iii. Sulfonylurea iv. NSAIDs v.Thiazolidinedione vi. Health care adjustment	i. Intake mean and median duration − 218 days and 240 days ii. Daily dose mean and median metformin − 1500 mg	i. Multivariate model, any metformin use - 15% reduced odds of CRC (AOR 0.85, 95% CI, 0.760.95) ii. Adjustment health care + metformin - 12% reduced odds of CRC (AOR 0.88, 95% CI 0.77–1.00)	Not mentioned	Not mentioned	No significant association with metformin dose, duration, or total exposure
920 diabetic patients with CRC Age 70.2 ± 8.6 years 63.3% male, 36.7% female^120^	YES	i. statins ii. insulin iii. Sulfonylurea iv. NSAIDs	Not mentioned	i. Extensive use –increased CRC risk, OR 1.43, 95% CI 1.08–1.90 ii. Significant increased risk in men, OR 1.81, 95% CI: 1.25–2.62 Ko0l	Not mentioned	Not mentioned	Extensive metformin intake increased risk of CRC
i. 675 with Type 2 DM from 1999 to 2009 ii. Age: 5 patients < 50, 269 patients between 50 and 69 and 401 patients above 70 iii. 437 males and 238 females iv. Stage I – IV of CRC^119^	Within the first 6 months of exposure	i. 6 months within CRC diagnosis - 88.7% surgery 28% chemotherapy 15% radiotherapy ii.63.1% sulfonylureas iii. 23.1% insulin	Metformin used after CRC diagnosis; Follow up – i. 6 months after diagnosis until death ii. within 5 years up to 14 years	i. HR 1.06, 95% CI 0.80, 1.40 ii.	i. cancer-specific mortality ii. HbA1C levels	Lesser incidence of congestive heart disease, myocardial infarction and peripheral vascular disease	No protective association between metformin use and CRC mortality

## Molecular mechanisms of metformin in CRC

### Metformin targets mTOR through AMPK and insulin/insulin-like growth factor (IGF) pathways

**Fig. 1:**
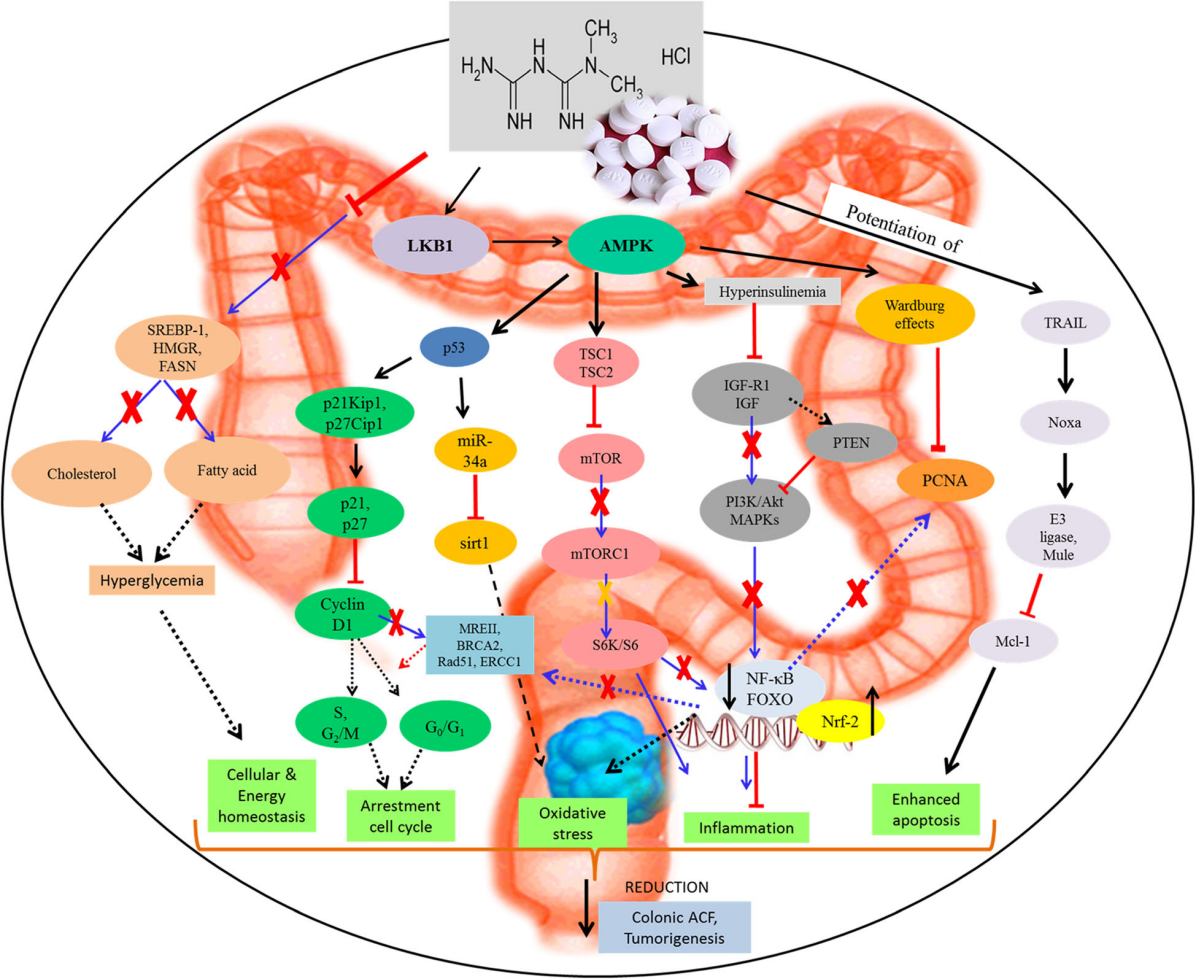
The anticancer molecular mechanisms mediated by metformin through the modulation of AMPK and cellular energy homeostasis. Metformin mainly modulates AMPK activation through LKB1 which activates and/or inactivates various downstream signalling targets such as mTOR, PTEN/PI3K-Akt, MAPKs, transcription factors (NF-κB, FOXO) and p53. The activation of these signalling pathways induce oxidative stress, apoptosis and cell cycle arrestment that inhibited formation of ACF and tumorigenesis in the colon cancer cell while suppressing cellular inflammation that is responsible to promote cell proliferation. The signalling activation or inhibition mediated by metformin is denoted by the red arrows and inhibition arrows, reversing the tumorigenesis mechanism indicated by the blue arrows

The pathogenesis of CRC is linked with multiple genetic alterations such as oncogenic Ras activation, hyperactivation of PI3K-Akt, p53 mutation, and dysregulation of Wnt pathway. Metformin is reported to interfere with the CRC cell growth, proliferation, and angiogenesis by rendering cell death via multifarious signaling pathways (Fig. 1) [61]. Various in vitro and in vivo studies have documented that metformin induces anticancer effect mainly by mediating 5’adenosine monophosphate (AMP)-activated protein kinase (AMPK)/mammalian target of the rapamycin (mTOR) pathway and insulin/ insulin-like growth factor-related pathways that modulate inflammation and inhibit colon tumor development and growth [31, 37, 56, 57]. Generally, metformin induces its anticancer effect via two main mechanisms: [1] direct mechanism resulting from its suppression of adenosine triphosphate (ATP) production due to the inhibition of mitochondrial complex I and [2] indirect mechanism involving “endocrine-type effects” related to its insulin-lowering activity which may suppress tumor development in hyperinsulinemic patients.

In the direct route, metformin activates AMPK, a major metabolic sensor involved in regulating cellular energy homeostasis. The activation of AMPK is mediated by other proteins including the enzymes liver kinase B1 (LKB1) (i.e., the serine-threonine kinase STK11), calcium/calmodulin-dependent protein kinase (CaMKK), and TGF-β-activated protein kinase 1 (TAK1). Metformin inhibits complex 1 of mitochondrial electron transport chain and thereby attenuates oxidative respiration resulting in ATP/AMP ratio imbalance, which in turn activates LKB1 and AMPK [122]. Following AMPK activation, metformin can induce the activation and inactivation of an array of upstream and downstream molecular signaling pathways that promote cell death. For example, treatment with metformin suppresses the development of intestinal polyp in Apc^Min/+^ mice by phosphorylating AMPK that suppresses mTOR/S6K/S6 signaling pathway [55]. The induction of AMPK can further induce the subsequent activation of tuber sclerosis complex/tuberin-2 (TSC2), an inhibitor of mTOR pathway that is cardinal in the cellular protein translational machinery and cell proliferation [87]. mTOR which possesses significant roles in cell growth and proliferation, apoptosis, inflammation, autophagy, and cytoskeletal organization can be found in two cellular complexes, termed mTOR complex 1 (mTORC1) and mTOR complex 2 (mTORC2) [123]. The mTORC1 is mainly characterized by the presence of raptor (regulatory-associated protein of mTOR) while mTORC2 is defined by the presence of rictor (rapamycin-insensitive companion of mTOR) [112]. Additionally, the activation of LKB1, can induce AMPK catalytic subunit phosphorylation even though LKB1 is not the main target protein of metformin [124]. Nonetheless, the activation of LKB1/AMPK/TSC2 pathway by metformin is extremely vital in suppressing the hyper-proliferation of CRC cells through dysregulated mTOR pathway. Additionally, without the presence of TSC2, metformin-activated AMPK can suppress mTOR/mTORC1 through phosphorylation of the raptor component of the mTORC1 complex [125]. Hosono et al. [86] demonstrated that metformin (250 mg/kg/day) for 6–32 weeks inhibited aberrant crypt foci (ASF) and colon polyp formation by inducing the activation of LKB1 and mTOR-dependent AMPK.

In the indirect mechanisms, metformin exerts its anticancer effect through the insulin/insulin-like growth factor-1 (IGF-1) pathway. In normal cells, the receptors for IGFs and insulin are widely expressed and can be phosphorylated following binding to its ligand which lead to the concomitant activation of downstream pathways such as PI3K/Akt/mTOR and RAS/RAF/mitogen-activated protein kinase (MAPK) pathways. The activation of these pathways via circulated insulin stimulates IGF-1/IGF-1R activation that promotes the initial tumor proliferation and growth. However, as an antidiabetic drug, metformin can promote the phosphorylation of IGF-1R that inhibits IGF-1 signalling which increases peripheral insulin sensitivity and muscle uptake of glucose while reducing plasma insulin levels and hepatic glucose output. As a result, the activation of IGF-1/IGF-1R is further inhibited leading to the indirect anti-proliferative effect of the cancer cell. For example, Cho et al. [37] demonstrated that the combination of metformin and CB-PIC enhances phosphorylation of ACC, AMPK훼 and pERK which suppresses mTOR and Akt activation in hypoxic SW620 cells. More importantly, the direct and indirect anticancer mechanisms of metformin are similar, as they both modulate mTOR as a common signaling target. These signaling pathways modulated by metformin are summarized in Fig. 1 in relation with other upstream and downstream mediators.

### Metformin induces apoptosis and autophagy through oxidative stress, inflammation, and metabolic homeostasis via AMPK and mTOR

The activation of AMPK by metformin is a cardinal step that modulates various transcription factors such as NF-κB and FOXO which regulates cellular apoptosis, oxidative stress, inflammation, and neoplastic malignancy. Metformin through its anti-inflammatory and anti-oxidant properties targets various cellular mechanisms responsible in the development of cancer that is associated with diabetes and obesity. Moreover, metformin enhances cellular apoptosis in CRC cells by modulating the production of anti- and pro-inflammatory mediators. Metformin inhibits IκBα degradation which suppresses expression of IL-8 and NF-κB activation in TNF-α-stimulated COLO 205 cells [59]. Moreover, metformin induces anti-inflammatory property that inhibits DSS-induced IκB kinase activation and reduced colitic cancer development in IL-10^−/−^ mice by augmenting AMPK activation in the intestinal epithelial cells. In addition, co-administration of metformin and DMH in Balb/c female mice effectively reduces the formation of AC and ACF (58.3 and 47.4%, respectively) through the modulation of oxidative stress and inflammation [126]. Metformin also upregulates p53 and Nrf2 expression while inactivating NF-κB which induces cellular apoptosis and modulation of oxidative stress and inflammation. The observations are corroborated by the reduction of malondialdehyde (MDA), inhibition of iNOS expression that decreased NO and nitrotyrosine, suppression of IL-10 and elevation of IL-1β.

Saber et al. [40] demonstrated that metformin in combination with 5-ASA suppresses the pro-inflammatory mediators such as IL-1β, IL-6, COX-2 and TNF-α, TNF-R1 and TNF-R2 which inactivates of NF-κB and STAT3. These molecular events further decreases MMP-2 and -9 expression and thus, suggests metformin capability to reduce the CRC cell proliferation, migration, and invasiveness. Furthermore, the suppression of NF-κB activation enhances apoptosis by reducing the Bcl-2 protein expression. Exposure to subtoxic concentration of metformin (2.5–10 mM) significantly potentiated the apoptosis inducing effect of tumor necrosis factor (TNF)-related apoptosis-inducing ligand (TRAIL) through Mcl-1 degradation in HCT116, HT29, DLD-1 and Colo25 cells [127]. Metformin in combination with TRAIL induced the dissociation of Noxa from Mcl-1 followed with an increased E3 ligase Mule activity that promoted polyubiquitination of Mcl-1 in the cancer cells. Another study reported that treatment with metformin alone or in combination with silibinin induced the expression of p-AMPK which suppressed mTOR phosphorylation and induced the activation of PTEN that inactivated PI3K-Akt. Furthermore, modulation of both AMPK/mTOR and PTEN/PI3K-Akt pathways increase the expression of cleaved caspase-3 and apoptosis inducing factor that promoted apoptosis in COLO 25 cells [38]. In a different study, the synergistic anticancer effects of metformin and vitamin D3 activated the AMPK(IGFI)/mTOR pathway that suppressed S6P expression and thus, inhibited the formation of early colon neoplasia rats and mice models [64]. It is reported that metformin significantly potentiates the vitamin D3 suppression of c-Myc and cyclin D1 mediated through via vitamin D receptor/β-catenin pathway.

Metformin regulates the energy and metabolic homeostasis by regulating the expression of key regulatory lipid enzymes that are associated in metabolic reprogramming of cancer cells through upstream kinase LKB1. Metformin through LKB1 activates AMPK which suppresses the expression of lipogenic transcription factor sterol regulatory element-binding protein-1 (SREBP-1) and its downstream targets such as fatty acid synthase (FAS) and 3-hydroxy-3-methyl glutaryl-CoA reductase [57, 128, 129]. Since this process is essential in regulating the metabolic homeostasis and thus, it modulates the plasma concentrations of glucose, insulin, triglycerides, and cholesterol. Metformin suppresses the effect of high-energy diet in promoting the growth of tumor in xenografts mice model (MC38 colon carcinoma cells) by reducing the insulin level and FASN while inactivating the Akt protein. Additionally, metformin induces apoptosis via the cleavage of poly (ADP-ribose) polymerase (PARP) via AMPK activation, inactivation of acetyl-CoA carboxylase and upregulation of BCL2/Adenovirus E1B 19 kDa Interacting Protein 3 (BNIP3) expression which ultimately suppressed tumor growth and volume [57]. Other than modulating survival and AMPK pathways, metformin also inhibits DMH-induced CRC in diabetic Sprague Dawley rats by reversing the Warburg effect [58] leading to suppression of ACF formation and reduction of PCNA expression, proliferation index of colonic tissues which decreases tumors volume. Metformin is also beneficial in treating inflammatory bowel disease (IBD) and the chronic or long-term IBD can induce the development of colitis-associated colon cancer (CAC). Jie et al. [58] suggested that metformin inhibition of the colon cancer cell and produced synergistic colon cancer-preventative effect in diabetic patients by modulating the expression of PKM2 and IDH1, two main isoenzymes involved in glycolysis and TCA cycles. The modulation of apoptosis in CRC models by metformin through oxidative stress, inflammation and metabolic homeostasis is further exemplified in Fig. 1 in relation with relevant signaling pathways.

### Metformin modulates cell cycle and p53 regulation

The modulation AMPK by metformin alters the cell mitosis since phosphorylated AMPK is found at the centrioles during the initial stage of cell cycle as well as in the constriction ring during the final stages of mitosis kinesins, tubulins, histones, auroras, and polo-like kinases. Moreover, this alteration of cell cycle is also dependent on the status of p53 as a transcription factors that regulates cell cycle arrestment, DNA repair, programmed cell death, and senescence [130, 131]. The p53 modulates mTOR by direct modulation AMPK and TSC2 as well as through the regulation PTEN transcription and activation of IGF-1/AKT pathways [132–134]. Cancer cells with a mutated p53 gene that are treated metformin are unable to reprogram their metabolism and therefore, rendered to undergo apoptosis. Metformin can induce cell cycle arrestment following the activation of LKB1/AMPK that activates p53 and inhibits mTOR. This activation of p53 is regulated by the suppression of cyclin D1 and expression of cyclin- dependent kinase inhibitors p27Kip1 and p21Cip1 [135]. For instance, metformin induces arrestment of cell cycle at G_0_/G_1_ phase via the inhibition of cyclin D1 expression and telomerase activity [32]. The activation of p53 induces the transcription of p21 which increases the expression of apoptotic genes leading to DNA-damage and fragmentation as well as G_0_/G_1_ arrestment. Additionally, metformin in combination with other chemotherapy drug can suppress cancer cell proliferation by regulating cell cycle differently. For example, Zhang et al. [45] demonstrated that pretreatment with metformin followed by 5-FU inhibited the proliferation of the SW620 cells by reducing the S phase population without altering the G_0_/G_1_ or G_2_/M phase. Furthermore, metformin can radiosensitize p53-deficient HCT116 cells by arresting the G_2_/M phase via suppression the DNA repair proteins such as MRE11, BRCA2, Rad51, and ERCC1 [48].

Metformin also inhibits CRC cells proliferation by regulating the expression of microRNAs that further modulate various signaling pathways. Feng et al. [47] demonstrated that suppression of HCT-116 cells proliferation and migration by metformin and 5-FU can be potentiated by knocking down miR-21 expression which in turn increased the Sprouty2, tumor suppressor expression and PTEN. In a different study, treatment with metformin induced microRNA-34a to inactivate the Sirt1/Pgc-1α/Nrf2 pathway leading to increased susceptibility of wild-type p53 cancer cells towards oxidative stress and therapeutic agent in HCT116 cells [136]. Sirtuin 1 (Sirt1), an oncogenic protein promotes resistance against oxidative stress and modulates apoptosis through the deacetylation of its targets such as p53 and FOXO1. The latter can induces a positive-feedback loop through miR-34a that enhances the Sirt1 expression. Sirt1 is found to be overexpressed in human breast, colon, non-small-cell lung, and prostate cancer cells. Sirt1 has been suggested to induce an oncogenic effect in cells expressing wild-type p53 but a tumor-suppressive effect in mutated p53 cells. Although the report by Do et al. found that metformin enhanced apoptosis in the wild-type p53 HCT116 cells by increasing the p53 expression and miR-34a which downregulates Sirt1 expression and its subsequent downstream effectors, the role of Sirt1 in cancer particularly CRC is still debatable and requires further validation.

## Conclusions and future perspectives

The current review depicts the beneficial use of metformin from preclinical, epidemiologic, and clinical studies as potential chemotherapeutic and adjuvant agent for CRC with notable association with T2DM. Furthermore, the long history and clinical experience of metformin against various cancer cases simply rebranding it as a potential old drug to be repurposed as cheap and effective chemotherapeutic drug. Metformin use as a chemotherapeutic agent for CRC also varies but transcendent among gender, age, patients with or without CRC history or resurrection and treatment regimens as sole agent or adjuvant to existing chemotherapeutic drugs. The application of metformin for various cancer treatment particularly CRC requires further evaluation whether it is effective in preventing the CRC recurrence.

Most of the epidemiologic reports of metformin in CRC are mainly centred among diabetic patients and thus, did not fully justify its overall beneficial use among patients with or without diabetes mellitus. Additionally, the lack of different population within the same and/or different gender in previous reports also lead to bias and confounding analysis. One of the biggest hindrances in evaluating such primary endpoint would be the short follow-up period in the studies of CRC. This is based on the observation from various sporadic colorectal tumor patients [137] and comparative lesion sequencing [138] which reported the development of carcinoma from large adenoma to carcinoma could take approximately, 15 years. Therefore, clinical studies that focus on the late stage of CRC with longer duration of metformin intervention and include inclusion and/or exclusion of period of CRC diagnosis information would offer better view on the protective effect of metformin against CRC. Additionally, the lack of essential information such as HbA1c in patients, lifestyle factors (obesity, tobacco smoke and alcohol use) as well as dose and duration of exposure to metformin and/or other interventions in some reports (such in Cardel et al., 2014; Jain et al., 2016) could potentially limit the metformin-CRC relationship and thus, warrant a more systematic follow-up studies. Nevertheless, most of the recent findings in this review demonstrated that metformin is now found to be effective in preventing ACF formation, total polyps and adenoma recurrence incident among nondiabetic CRC patients. This highlights the multifarious positive potential of metformin as chemotherapeutic drug among different patients status and hence, providing the lead desired in managing the treatment of CRC and diabetes, simultaneously. Additionally, the reported marginal to mild side-effects of metformin further accentuate the chemopreventive potential and safer properties of metformin. Furthermore, the ability of metformin to treat diabetes through AMPK modulation that also induces anticancer effect associated with the activation and/or inactivation of various downstream targets illustrate the double therapeutics value of metformin. Therefore, up to this date, metformin is seen as a beneficial oral diabetic drug with vast chemotherapeutic potential against CRC.

A number of studies have reported some contradictory findings on metformin use in the management of CRC. The lack of CRC prevention among postmenopausal women and among specific population in UK and Germany further suggest that metformin use still requires more clinical and epidemiologic studies that encompasses more specific target groups. In addition, even though recent finding highlighted the ability of metformin to prevent ACF and CRC recurrence among Japanese population, however, more clinical trials with different target population are needed to further strengthen this result. In short, based on the various preclinical, epidemiologic and clinical studies, metformin, a beneficial metabolic drug of diabetes with pleotropic molecular targets, hold the substantial therapeutic value not only in the modulation of metabolic homeostasis but more importantly, as potential anti-neoplastic agent for CRC. However, extensive randomized clinical studies on large number of subjects will further strengthen the confirmation of the therapeutic effectiveness of metformin for the treatment of CRC.

## Data Availability

Not applicable.

## Abbreviations

ACF: Aberrant crypt foci
AICAR: 5-Aminoimidazole-4-carboxamide ribonucleotide
AMPK: 5′ AMP-activated protein kinase
APAF-1: Apoptotic protease activating factor 1
CAC: Colitis-associated colon cancer
CaMKK: Calcium/calmodulin-dependent protein kinase
CRC: Colorectal cancer
CRT: Chemoradiotherapy
DDD: Defined daily dose
DMH: 1,2-dimethylhydrazine dihydrochloride
DSS: dextran sodium sulfate
EMT: Epithelial mesenchymal transition
FAS: Fatty acid synthase
FASN: Fatty acid synthase gene
FuOx: 5-fluorouracil-oxaliplatin
HMGB1: High-mobility group box 1 protein
HR: Hazard ratio
IGF: Insulin growth factor
IR: Ionizing radiation
LCA: Litocholic acid
LKB1: Liver kinase B1
MAPKs: Mitogen-activated protein kinases
Map-LC3: Microtubule-associated protein 1 light chain 3
Mcl-1: Myeloid cell leukemia 1
Nrf-2: Nuclear factor E2-related factor 2
mTOR: Mammalian target of rapamycin
mTORC1: Mammalian target of rapamycin complex 1
mTORC2: Mammalian target of rapamycin complex 2
PARP: Poly (ADP-ribose) polymerase
PCNA: Proliferating cell nuclear antigen
PFS: Progression free survival
OR: Odd ratio
OS: Overall survival
RASSF1A: Ras asscociation domain family 1 isoform A
ROS: Reactive oxygen species
SREBP-1: Sterol regulatory element-binding protein-1
STK11: Serine-threonine kinase
TAK1: TGF-β-activated protein kinase 1
TGF-β: Transforming growth factor
TRAIL: Tumor necrosis factor (TNF)-related apoptosis-inducing ligand
TSC2: Tuber sclerosis complex/tuberin-2
T2DM: Type 2 diabetes mellitus
4-IPP: 4-iodo-6-phenylpyrimidin
5-ASA: to 5-aminosalicylic acid
5-FU: 5-fluorouracil
